# Combination of Mitochondrial and Plasma Membrane Citrate Transporter Inhibitors Inhibits De Novo Lipogenesis Pathway and Triggers Apoptosis in Hepatocellular Carcinoma Cells

**DOI:** 10.1155/2018/3683026

**Published:** 2018-01-09

**Authors:** Wan-angkan Poolsri, Phornpun Phokrai, Somrudee Suwankulanan, Narinthorn Phakdeeto, Pattamaphorn Phunsomboon, Dumrongsak Pekthong, Lysiane Richert, Sutatip Pongcharoen, Piyarat Srisawang

**Affiliations:** ^1^Department of Physiology, Faculty of Medical Science, Naresuan University, Phitsanulok 65000, Thailand; ^2^Clinical Research Unit, Faculty of Medicine, Naresuan University, Phitsanulok 65000, Thailand; ^3^Department of Pharmacy Practice, Faculty of Pharmaceutical Sciences, Naresuan University, Phitsanulok 65000, Thailand; ^4^KaLy-Cell, 20A rue du Général Leclerc, 67115 Plobsheim, France; ^5^Laboratoire de Toxicologie Cellulaire, Université de Bourgogne Franche-Comté, EA 4267, Besançon, France; ^6^Department of Medicine, Faculty of Medicine, Naresuan University, Phitsanulok 65000, Thailand; ^7^Centre of Excellence in Medical Biotechnology, Faculty of Medical Science, Naresuan University, Phitsanulok 65000, Thailand; ^8^Center of Excellence in Petroleum, Petrochemicals and Advanced Materials, Faculty of Science, Naresuan University, Phitsanulok 65000, Thailand

## Abstract

Increased expression levels of both mitochondrial citrate transporter (CTP) and plasma membrane citrate transporter (PMCT) proteins have been found in various cancers. The transported citrates by these two transporter proteins provide acetyl-CoA precursors for the de novo lipogenesis (DNL) pathway to support a high rate of cancer cell viability and development. Inhibition of the DNL pathway promotes cancer cell apoptosis without apparent cytotoxic to normal cells, leading to the representation of selective and powerful targets for cancer therapy. The present study demonstrates that treatments with CTP inhibitor (CTPi), PMCT inhibitor (PMCTi), and the combination of CTPi and PMCTi resulted in decreased cell viability in two hepatocellular carcinoma cell lines (HepG2 and HuH-7). Treatment with citrate transporter inhibitors caused a greater cytotoxic effect in HepG2 cells than in HuH-7 cells. A lower concentration of combined CTPi and PMCTi promotes cytotoxic effect compared with either of a single compound. An increased cell apoptosis and an induced cell cycle arrest in both cell lines were reported after administration of the combined inhibitors. A combination treatment exhibits an enhanced apoptosis through decreased intracellular citrate levels, which consequently cause inhibition of fatty acid production in HepG2 cells. Apoptosis induction through the mitochondrial-dependent pathway was found as a consequence of suppressed carnitine palmitoyl transferase-1 (CPT-1) activity and enhanced ROS generation by combined CTPi and PMCTi treatment. We showed that accumulation of malonyl-CoA did not correlate with decreasing CPT-1 activity. The present study showed that elevated ROS levels served as an inhibition on Bcl-2 activity that is at least in part responsible for apoptosis. Moreover, inhibition of the citrate transporter is selectively cytotoxic to HepG2 cells but not in primary human hepatocytes, supporting citrate-mediating fatty acid synthesis as a promising cancer therapy.

## 1. Introduction

Hepatocellular carcinoma (HCC) is a principal common global cause of cancer deaths and the fifth most frequent malignancy in patients with cirrhosis. The incidence of HCC is the highest observed in South East Asia, including Thailand [1]. The earliest studies focused on cancer cell biology of which the signaling pathways caused uncontrolled proliferation. However, in recent years, more evidence has shown that reprogramming metabolism can be an important process during tumorigenesis [2, 3]. The reprogramming of energy pathways in cancers, switching the major metabolism pathway from oxidative phosphorylation (OXPHOS) to rely on aerobic glycolysis, is known as the Warburg effect [4, 5]. This hallmark feature promotes increased glucose uptake and intermediate flux for de novo synthesized biomolecules, including nucleotide, amino acids, and lipids to support high tumor proliferative and progression rate phenotypes of cancer [6, 7]. Intermediates from OXPHOS are redirected into the de novo lipogenesis (DNL) pathway to provide precursors for long chain fatty acids (LCFAs) synthesis prevailing in cancer cells while for most normal cells their lipids come from the abundant levels in the circulation. The enzymes participating in the DNL pathway are upregulated or constitutively expressed in most types of cancer cells [8]. High intracellular level of monounsaturated fatty acids (MUFAs) activates lung cancer development and progression [9]. Suppression of de novo fatty acid synthesis enhances apoptosis in cancer cells without exerting a cytotoxic effect on normal cells, suggesting DNL as a target for selective and effective cancer therapies in several cancer models [10–15]. The DNL pathway uses cytosolic citrate exported from mitochondria and transported from circulation into the cytoplasm which is then converted to acetyl-CoA by ATP-citrate lyase (ACLY), followed by carboxylation to form malonyl-CoA by acetyl-CoA carboxylase (ACC). Fatty acid synthase (FASN) uses acetyl-CoA, malonyl-CoA, and NADPH to elaborate LCFAs, especially 16-C palmitate. LCFAs are then metabolized through fatty acid *β*-oxidation and desaturated to MUFAs, leading to promotion of cell proliferation. Anticancer therapy targeting the DNL enzymes has been extensively studied to become one of the most efficient therapies by promoting cancer cell apoptosis without affecting nontransformed cells [11].

Two sources of intracellular citrate are transported by protein transporters, including the mitochondrial citrate transport protein (CTP) or SLC25A1 or citrate carrier (CiC) and plasma membrane citrate transporter protein (PMCT) or SLC13A5 or Na^+^-dependent citrate transporter (NaCT). CTP located on the inner membrane of mitochondria plays a pivotal role in connecting intermediary metabolisms of carbohydrate catabolism and lipogenesis by exporting acetyl-CoA in the form of citrate exchanged with malate from mitochondria to cytosol. CTP mRNA and protein levels are found to be abundantly expressed in human liver, where their activity has been reported to involve a particularly high fatty acid synthesis [16, 17]. Other tissues have been found, including pancreas and kidney, but low expression or absence has been reported in brain, heart, skeletal muscle, placenta, and lung tissue [17]. Overexpression of CTP has been reported in human colorectal cancer HT29 and Colo205 cells [18]. Kanplan et al. suggest that citrate flux from mitochondria TCA cycle into cytoplasm has higher in Morris hepatoma 3924A and hepatoma 16 than normal liver cells [19]. Supporting data obtained from web database of Human Protein Atlas (http://www.proteinatlas.org) has reported a high expression level of SCL25A1 or CTP in liver cancer cells. Consistent evidence supporting a regulatory role of CTP on cancer proliferation and progression has been reported showing the anticancer effects of CTP inhibition in xenograft of breast cancer and lung cancer [20]. However, the mechanism of cytotoxicity of CTP inhibition as an attractive target for a promising anticancer therapy is still unidentified.

Citrate is also imported by PMCT called a Na^+^/citrate transporter or sodium-coupled citrate transporter, NaCT, from an extracellular circulation citrate across the plasma membrane into the cytoplasm [21]. NaCT identified recently in mammals is expressed predominantly in the liver [22]. NaCT is structurally and functionally related to the product of the* Indy* (I am not dead yet) gene in* D. melanogaster* and NAC-2 in* C. elegans* [23]. Dysfunction of these genes exhibits lifespan extension, decreases body size, and reduces fat content [24, 25]. Supporting this report, depletion of NaCT reduces hepatic lipid production and plasma glucose levels in high fat diet animals [26], and reduction of PMCT expression reduces fatty acid content associated with improved insulin sensitivity and prevented diet-induced nonalcoholic fatty liver disease (NAFLD) in adult C57BL6/J mice [27]. There is a correlation of cancer development and NAFLD [28, 29]. It has also been shown that the inflammatory response in adipose tissues is promoted by lipid accumulation upon cytosolic citrate fluxed from mitochondrial source and enhanced by citrate exogenously uptake [30]. Thus, inhibition of PMCT appears to be a candidate therapeutic target of NAFLD-induced cancer. Data obtained from web database of Human Protein Atlas (http://www.proteinatlas.org) has reported a high expression level of human SCL13A5 or PMCT protein in liver cancer cells. Recent report from a knockdown experiment of PMCT suggests a significant antiproliferation effect on hepatoma HepG2 and HuH-7 cells via the mechanism involving a decreased intracellular levels of ATP/ADP ratio [31]. However, the proposed mechanism of PMCT inhibition on antioncogenic properties needs further experiments.

Thus, the present study was performed to identify apoptotic induction of CTP and PMCT inhibition in hepatocellular carcinoma cells, HepG2 and HuH-7. Decreased intracellular citrate level was hypothesized to cause inhibition of the DNL pathway leading to reduction of cell viability and finally induction of apoptosis in hepatocellular carcinoma cells. Interestingly, our result showed that a combination treatment generates a more potent cytotoxic effect than either inhibitor alone. Combined CTPi and PMCTi treatment showed a greater cell viability suppression effect in HepG2 cells than HuH-7 cells. A strong inhibition of intracellular citrate accumulation from combined transport inhibitors produced depletion of most of fatty acid synthesis. Moreover, synergistic anticancer efficiency of combined CTP and PMCT inhibition was aimed to lower doses used of each inhibitor. The results obtained from the present study may provide an opportunity for further development of more potential cancer therapy as well as cancer prevention caused by metabolic disorders.

## 2. Materials and Methods

### 2.1. Primary Cell and Cell Line Culture

Human hepatocellular carcinoma cells, HepG2 cells, were purchased from the American Type Culture Collection (HB-8065; ATCC, Manassas, VA, USA) and HuH-7 cells were purchased from Japanese Collection of Research Bioresources Cell Bank (JCRB0403; JCRB, Osaka, Japan). HepG2 cells were cultured in Eagle's Minimum Essential Medium (EMEM) (Corning, Manassas, VA, USA). HuH-7 cells were cultured in Dulbecco's modified Eagle's medium (DMEM) (Corning, Manassas, VA, USA). The Medium was supplemented with 10% fetal bovine serum (FBS) (Gibco, MA, USA) and 1% of 100 IU/ml penicillin and 100 *μ*g/ml streptomycin (Gibco, MA, USA). The culture was routinely maintained in a 5% CO_2_ with 95% humidified incubator at 37°C. Primary human hepatocytes were kindly provided by Professor Dr. Lysiane Richert, Scientific Director KaLy-Cell, 20A, rue du Général Leclerc, 67115 Plobsheim, France. Cells were cultured in Human Hepatocyte Maintenance Medium (Primacyt, Schwerin, Germany) containing 0.1 M dexamethasone (DEX), 0.1% of 100 IU/ml penicillin and 100 *μ*g/ml streptomycin, 4 mg/l insulin, and 1% fetal bovine serum. Cells were allowed to attach by incubating under a 5% CO_2_ air 95% humidified atmosphere maintained at 37°C.

### 2.2. MTT Assay for Detection of Cell Viability

After 24 h of plating, cells were incubated with corresponding concentrations of 4-chloro-3-[(3-nitrophenyl) amino] sulfonyl benzoic acid (mitochondrial citrate transport protein inhibitor or CTPi) (ChemBridge Corporation #6652048), 2-(benzylsulfanyl)-N-[(pyridin-2-yl) methyl] propanamide (plasma membrane citrate transporter inhibitor or PMCTi) (TimTec ID ST056138) and the combination of CTPi and PMCTi. After 24 h treatment, 3-(4,5-dimethylthiazol-2-yl)-2,5-diphenyltetrazolium bromide (MTT) solution was added and incubated for 3 h. The formazan crystals were dissolved in DMSO and cell viability was then quantified by measuring absorbance at 595 nm using microplate reader (Synergy HT Multi-Mode, BioTek Instruments, Inc.).

### 2.3. Flow Cytometry Analysis for Detection of Apoptosis

The apoptotic cell was determined using Muse annexin V and dead cell assay kit (Merck Millipore, Germany). The assay is based on the detection of phosphatidylserine (PS) on the surface of apoptotic cells, using fluorescent labeled annexin V in combination with the dead cell marker, 7-AAD. Briefly, following exposure to inhibitors at the indicated IC_50_ concentrations, cells were harvested and the reagent from the assay kit was added; apoptotic stained cells were detected by Muse cell analyzer (Merck Millipore, Germany).

### 2.4. Analysis of Cell Cycle Arrest

The effect of citrate transporter inhibitors on cell cycle progression was determined using the Muse Cell Cycle assay kit (Merck Millipore, Germany). The assay is based on staining cells with PI to analyze DNA contents. Analysis of cellular DNA was performed by Muse Cell Analyzer.

### 2.5. Determination of Mitochondrial Membrane Potential (ΔΨ*m*)

The loss of ΔΨm was assessed by flow cytometry using JC-1 dye (5,5′,6,6′-tetrachloro-1,1′,3,3′-tetraethylbenzimi-dazolylcarbocyanine iodide) (Life Technologies, Thermo Scientific, NY, USA), a mitochondrial membrane potential probe which enters selectively in mitochondria. The JC-1 aggregated forms accumulate inside mitochondria in response to changing of ΔΨm. A healthy mitochondrion in polarized state exhibits red fluorescence emission with high aggregates; JC-1 forms in the mitochondrial matrix while it remains in the monomeric form in the cytoplasm with a depolarized state of the mitochondrial membrane and exhibits green fluorescence emissions. After 24 h of treatment, cells were harvested and incubated with JC-1 dye at 37°C and 5% CO_2_ for 45 min. The disruption of ΔΨm was detected by FACSCalibur flow cytometry, and the data were analyzed using CellQuset Pro software.

### 2.6. Western Blotting for Detection of Protein Expression

To determine protein expression in the DNL pathway, total protein extraction was performed by M-PER (Mammalian Protein Extraction Reagent) (Thermo Fisher Scientific Inc., Rock ford, IL, USA). Extracted protein concentration was quantified by BCA assay (Bicinchoninic acid; Thermo Scientific, Rock ford, IL, USA). Extract proteins were separated by 8–12% SDS polyacrylamide gel electrophoresis and then immunoblotted with specific primary antibodies, including anti-fatty acid synthase (FASN; 1 : 1000) (Abcam, Biomes Diagnostic Co., Ltd., Thailand), anti-Acetyl-CoA carboxylase (ACC; 1 : 1000) (Merck Millipore, Germany), anti-ATP-citrate lyase (ACLY; 1 : 1000), and anti-*β* actin (1 : 800) (Cell Signaling Technology Inc., USA). Then, immunoblots were incubated with horseradish peroxidase-conjugated goat anti-Rabbit IgG secondary antibody (Life Technologies, Invitrogen, NY, USA) followed by visualization with the enhanced chemiluminescence HRP substrate (Luminate Forte, Merck Millipore, Germany). The intensity of protein bands was measured using CCD camera (ImageQuant LAS 4000).

### 2.7. Measurement of Intracellular Citrate Level

Cytosolic citrates are substrates for the DNL pathway. Conversion of citrate to pyruvate was quantified by the citrate bioAssay kit (C5802: US Biological; Life Sciences, Salem, MA, USA) as described in the manufacturer's protocol. The fluorescence of pyruvate product was measured at Ex/Em 535/590 nm by a microplate reader.

### 2.8. Measurement of Intracellular Long Chain Free Fatty Acid Level

To determine intracellular long chain free fatty acid level, a product from the DNL pathway, the free fatty acid bioAssay kit (F0019-94; US Biological; Life Sciences, Salem, MA, USA), was used as described in the manufacturer's protocol. In the assay, long chain free fatty acids are converted to CoA derivatives and then oxidized with concomitant generation of fluorescence and detected by fluorometry at Ex/Em 535/590 nm with a microplate reader.

### 2.9. Determination of Carnitine Palmitoyl Transferase-1 (CPT-1) Activity

CPT-1 activity was quantified by a spectrophotometric method as described previously [32]. Briefly, following 24 h of treatment, mitochondrial proteins were isolated by lysis buffer (Tris-HCl pH 7.4) containing 0.25 mM sucrose and 1 mM EDTA. The mitochondrial protein content was determined and added the reaction mix containing Tris-buffer (100 mM, pH 8.0, 0.1% Triton-X-100, 1 mM EDTA), 0.01 mM palmitoyl-CoA, and 0.5 mM DTNB. After adding L-carnitine 1.25 mM, sample was measured O.D. at 412 nm using a microplate reader.

### 2.10. Measurement of Bcl-2 Activation

Bcl-2 activity was measured by flow cytometry using the Muse Bcl-2 activation dual detection kit (MCH200105; EMD Millipore, Germany) as described in the manufacturer's protocol and detected by Muse Cell Analyzer.

### 2.11. Determination of Reactive Oxygen Species (ROS)

ROS production was assessed using 5-(and-6)-chloromethyl-2′, 7′-dichlorodihydrofluorescein diacetate, acetyl ester (C6827; CM-H2DCFDA; Molecular Probe, Thermo Fisher Scientific Inc., MA, USA), which is an indicator of ROS production. Intracellular ROS level was detected by the converting of H2DCFDA to a DCF that exhibits an orange fluorescence by oxidation and removal of acetate groups by cellular esterases. Cells were analyzed by FACSCalibur flow cytometry with CellQuset Pro software.

### 2.12. Statistical Analysis

The results were analyzed by *t*-test and one-way ANOVA using a Turkey test as a post-test. *p* < 0.05 versus the control was considered statistically significant using Graph Prism Software, version 5. Three independent experiments were performed for statistical analysis and expressed as mean ± SEM.

## 3. Results

### 3.1. Citrate Transporter Inhibitors Decreased Viability of HCC Cells

**Figure 1:**
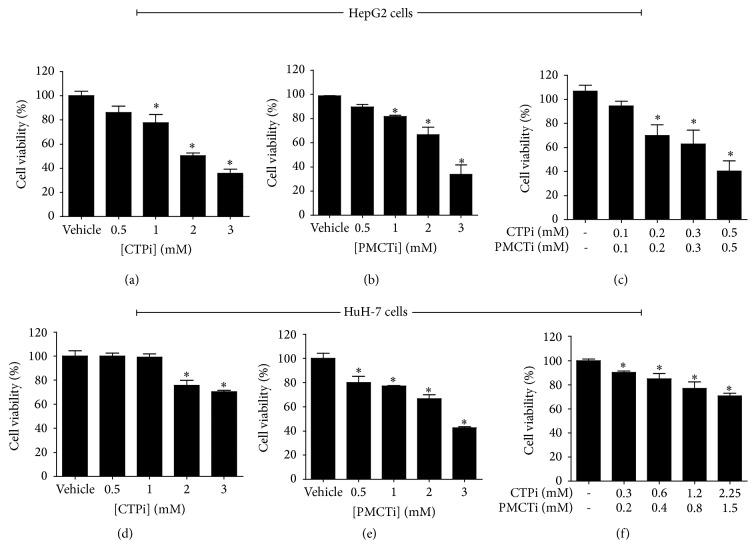
Effect of citrate transporter inhibitors on the viability of HCC cells. Cells were treated with different concentrations of citrate transporter inhibitors for 24 h, (a)–(c) HepG2 cells and (d)–(f) HuH-7 cells. MTT assay was performed and expressed an inhibition as a percentage of cell viability compared with 100% of the vehicle control. Three independent experiments were performed for statistical analysis and expressed as mean ± SEM. ^**∗**^*ρ* < 0.05 versus the control group.

Treatments of HepG2 and HuH-7 cells with CTPi, PMCTi, and the combination of CTPi and PMCTi resulted in decreased cell viability in a dose-dependent manner (Figures 1(a)–1(f)). All the inhibitors most potently inhibited HepG2 cells and showed better cytotoxic efficacy with IC_50_ value approximately of 2.5 mM of CTPi or PMCTi in comparison to HuH-7 treated cells which had IC_50_ value approximately of 4.5 mM of CTPi and 3 mM of PMCTi. In HepG2 cells, the combination treatment showed a greater cytotoxic effect than CTPi or PMCTi treated individually with IC_50_ approximately of 0.4 mM of each inhibitor (Figure 1(c)). The combined treatment exhibited synergistic inhibition of cell viability in HepG2 cells. Our results also showed that a combination treatment in HuH-7 cells generated a more effective cytotoxic effect than either inhibitor alone. This study suggests that relatively lower concentration of combined CTPi and PMCTi promoting cytotoxic effect against HepG2 cells as compared with HuH-7 cells represents a potent cancer suppression effect.

### 3.2. Citrate Transporter Inhibitors Induced Apoptosis in HCC Cells

**Figure 2:**
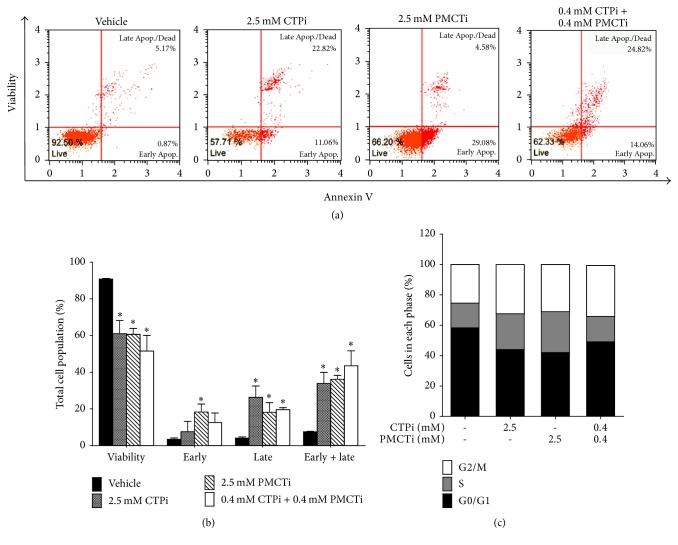
Effect of citrate transporter inhibitors on apoptosis and cell cycle arrest of HepG2 cells. Cells were treated with IC_50_ concentrations of CTPi, PMCTi, and a combination of 0.4 mM of each inhibitor for 24 h. The control was defined as a vehicle for which cells were treated with a medium containing 0.2% DMSO alone. (a) Flow cytometry shows representative dot plot analysis. (b) Effect of citrate transporter inhibitors on apoptosis of HepG2 cells are represented in the bar graph. (c) The bar charts show percentages of cells population in G0/G1, S, and G2/M phases (set as 100%). All data are expressed as mean ± SEM of at least three independent experiments. ^**∗**^*ρ* < 0.05 versus the control group.

**Figure 3:**
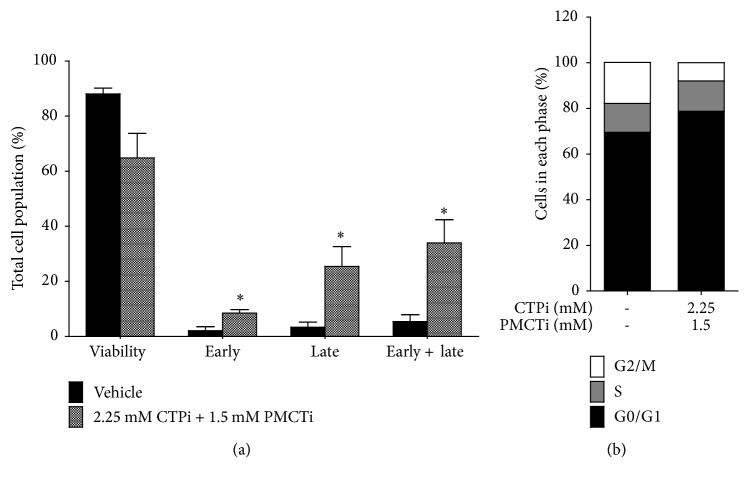
Effect of citrate transporter inhibitors on apoptosis and cell cycle arrest of HuH-7 cells. Cells were treated with the combination of half of IC_50_ of each inhibitor for 24 h. The control was defined as a vehicle for which cells were treated with a medium containing 0.2% DMSO alone. (a) Effect of both inhibitors on apoptosis of HuH-7 cells are represented in the bar graph. (b) The bar charts show percentages of cells population in G0/G1, S, and G2/M phases (set as 100%). All data are expressed as mean ± SEM of at least three independent experiments. ^**∗**^*ρ* < 0.05 versus the control group.

HepG2 cells were treated with CTPi, PMCTi, and combination of each inhibitor at IC_50_ concentrations obtained from the MTT result for 24 h. Because each inhibitor exhibited relative high IC_50_ concentration (>3 mM) against HuH-7 cells, the combination of half of IC_50_ value from each inhibitor was selected for further evaluation. Cell death with apoptosis was then detected. The results showed that CTPi or PMCTi therapy of 2.5 mM in HepG2 cells significantly reduced the viability of cells to 58–66% and increased apoptotic cell death rate to 34–36% compared with the control where apoptotic cells were 8% (Figures 2(a) and 2(b)). A potent apoptotic induction effect approximately 43% was shown in cells treated with a combination of CTPi and PMCTi at 0.4 mM of each inhibitor. Furthermore, apoptosis induction in HuH-7 cells was significantly increased to 34% compared with the control where apoptosis cells were 5% (Figure 3(a)). Thus, a combination with the lower concentration of CTPi plus PMCTi rather than the single inhibitor exhibited potent apoptotic induction in liver cancer cells.

### 3.3. Citrate Transporter Inhibitors Exhibited Cell Cycle Arrest in HCC Cells

To investigate cell cycle progression during apoptosis, HepG2 and HuH-7 cells were exposed with CTPi and PMCTi inhibitor. After 24 h, treated cells were detected in their population in each phase of the cell cycle. As shown in Figure 2(c), HepG2 cells treated with CTPi, PMCTi, and a combination of CTPi and PMCTi caused cell cycle arrest in S and G2/M phases with reduction of cells in G0/G1 phase to 44%, 42%, and 49%, respectively, compared with 60% of the vehicle. HuH-7 cells treated with a combination of CTPi and PMCTi exhibited cell cycle arrest in G0/G1 phase with reduction of cells in G2/M phase to 8% compared with 18% of the vehicle. Thus, Cell cycle arrest supports the apoptotic induction effect of citrate transporter inhibitors in HepG2 and HuH-7 cells, consistent with previous report [33].

### 3.4. Citrate Transporter Inhibitors Promoted Loss of ΔΨm in HepG2 Cells

**Figure 4:**
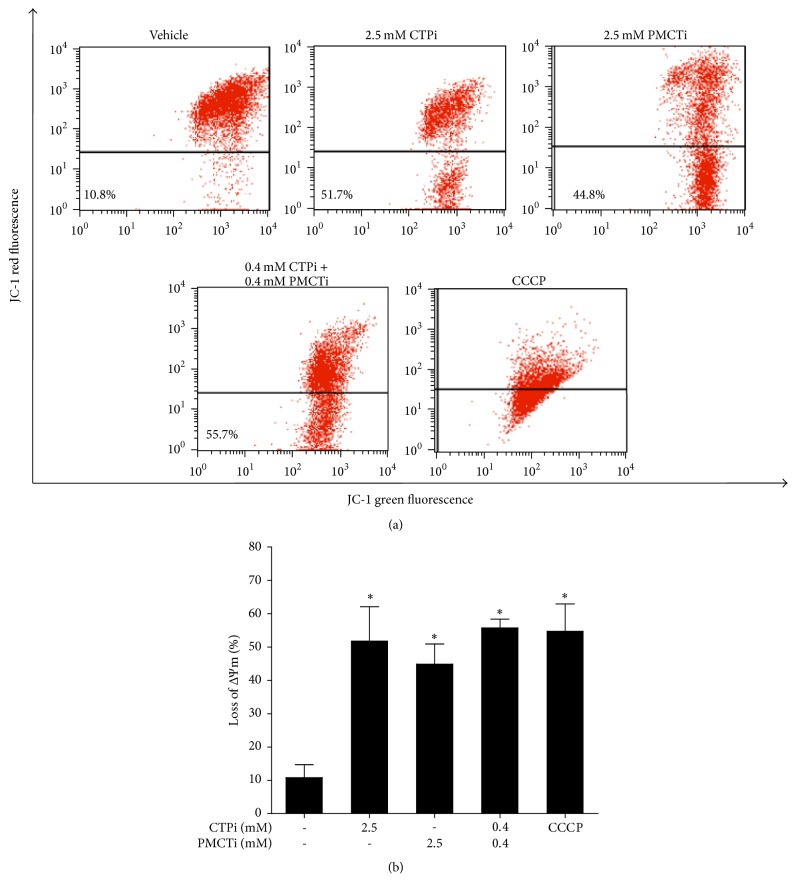
Effect of citrate transporter inhibitors on mitochondrial membrane potential (ΔΨm) in HepG2 cells. After 24 h, cells were incubated with CTPi, PMCTi, and the combination of 0.4 mM of each inhibitor with IC_50_ concentrations. (a) ΔΨm in HepG2 cells was detected by flow cytometry and is shown in representative dot plot analysis. (b) Percentage of the loss of ΔΨm is shown in a bar graph. The control was defined as a vehicle for which cells were treated with a medium containing 0.2% DMSO alone. The CCCP was used as a positive control of the loss of ΔΨm. Data from at least three dependent experiments are expressed as mean ± SEM, ^**∗**^*ρ* < 0.05.

JC-1 staining was performed to investigate the effect of the citrate transporter inhibitor on induction of apoptosis through the loss of ΔΨm. HepG2 cells treated with CTPi or PMCTi exhibited loss of ΔΨm to 51.7% and 44.8%, respectively following 24 h incubation with 2.5 mM of a single inhibitor (Figures 4(a)–4(b)). The combination of CTPi and PMCTi at 0.4 mM of each for 24 h exhibited a more potent effect on enhanced loss of ΔΨm to 55.7% compared with approximately 10.8% of the control (Figures 4(a) and 4(b)). This result suggests that citrate transporter inhibitors exert mitochondrial-dependent apoptotic induction in HepG2 cells.

### 3.5. Citrate Transporter Inhibitors Had No Apoptosis Induction Effect in Primary Human Hepatocyte Cells

**Figure 5:**
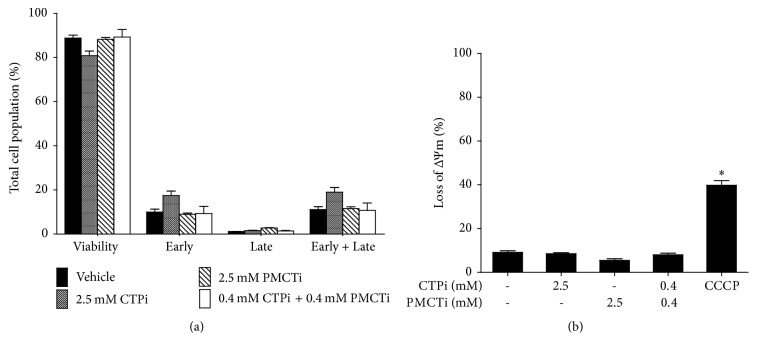
Effect of citrate transporter inhibitors on apoptosis and ΔΨm in primary human hepatocyte cells. Cells were treated with CTPi, PMCTi, and the combination of 0.4 mM of each inhibitor at IC_50_ concentrations of each for 24 h. (a) Apoptotic cells were detected by flow cytometry and are shown in representative bar graph analysis. (b) Percentage of the loss of ΔΨm in primary human hepatocytes is shown in a bar graph. The control was defined as a vehicle for which cells were treated with a medium containing 0.2% DMSO alone. The CCCP was used as a positive control of the loss of ΔΨm. Data from at least three dependent experiments are expressed as mean ± SEM, ^**∗**^*ρ* < 0.05.

Primary hepatocyte cells treated with 24 h incubation with 2.5 mM of a single citrate transporter inhibitors and a combination of each inhibitor did not exhibit apoptosis (Figure 5(a)) and dissipation of ΔΨm (Figure 5(b)). Thus, CTPi, PMCTi, and the combination of each inhibitor are selective compounds for treatment in liver cancer cells.

### 3.6. The Combination of Citrate Transporter Inhibitors Decreased Citrate and Free Fatty Acid Levels but Did Not Affect Lipogenic Protein Expression in HepG2 Cells

**Figure 6:**
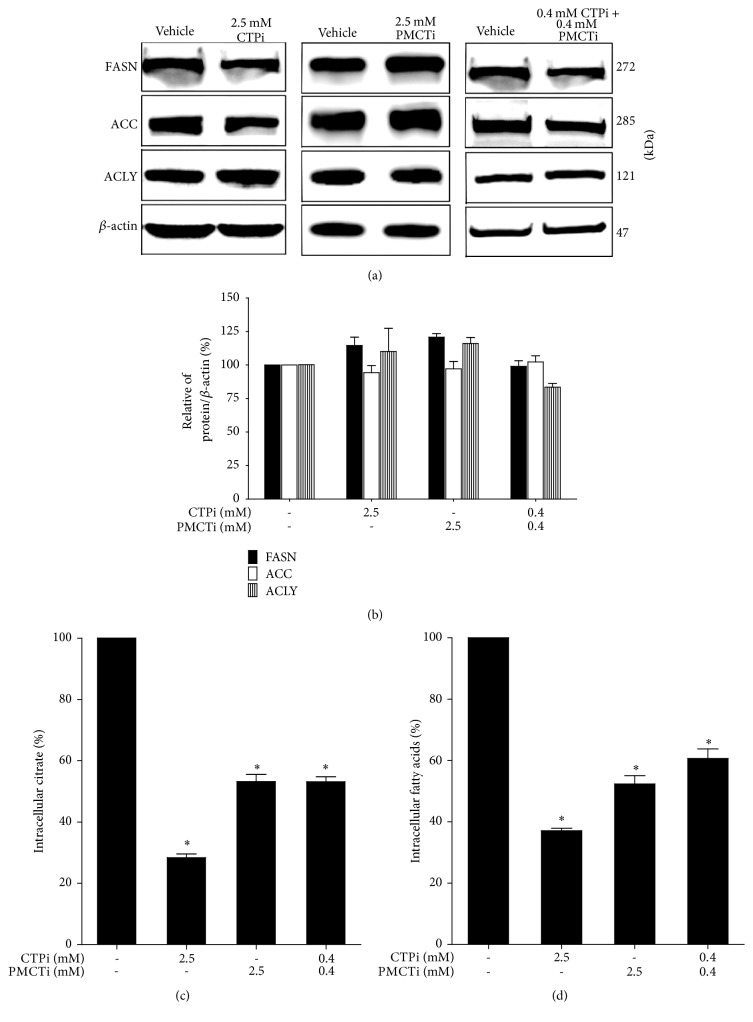
Effect of citrate transporter inhibitors on DNL pathway in HepG2 cells. Cells were treated with CTPi, PMCTi, and the combination of 0.4 mM of each inhibitor at IC_50_ concentrations of each for 24 h. The control was defined as a vehicle for which cells were exposed to a medium containing 0.2% DMSO alone. An equal amount of total protein extracts was subjected to an immunoblotting assay to detect expression of FASN, ACC, and ACLY proteins. *β*-actin was used to normalize equal amount and intensity of protein bands. (a) The expression of lipogenic proteins was detected by immunoblotting technique and visualized using a CCD camera. (b) Quantified density values were obtained and expressed as a percentage of *β*-actin/protein ratio. (c)-(d) Intracellular citrate and fatty acid levels were quantified as described in materials and methods. The data shows mean ± SEM. ^**∗**^*ρ* < 0.05 from three independent experiments.

We further investigated the decrease of fatty acid levels in the DNL pathway, noting inhibition of citrate transport could potentially exert apoptotic induction in HepG2 cells. Reduction of fatty acid synthesis is known to cause apoptosis in cancer cells as reported previously [34, 35]. The expression of proteins in the DNL pathway was investigated by immunoblot analysis. After cells were treated with CTPi, PMCTi, and the combination of 0.4 mM of each inhibitor for 24 h, the expression of proteins, including FASN, ACC, and ACLY did not alter (Figures 6(a) and 6(b)). Thus, each single inhibitor and combination of CTPi and PMCTi in HepG2 has no effect on DNL lipogenic protein expression in HepG2 cells. However, CTPi, PMCTi, and a combination of both inhibitors decreased intracellular citrate to 30%, 53%, and 52% and decreased intracellular fatty acid to 37%, 51%, and 60%, respectively, compared with 100% of the vehicle control (Figures 6(c) and 6(d)). These results demonstrate that combination of CTPi and PMCTi causes marked suppression of the DNL pathway in HepG2 cells.

### 3.7. Suppressed CPT-1 Activity as a Result of Decreased Fatty Acid Level by Combined CTPi and PMCTi Contributed to Apoptosis without Increase of Malonyl-CoA Level in HepG2 Cells

**Figure 7:**
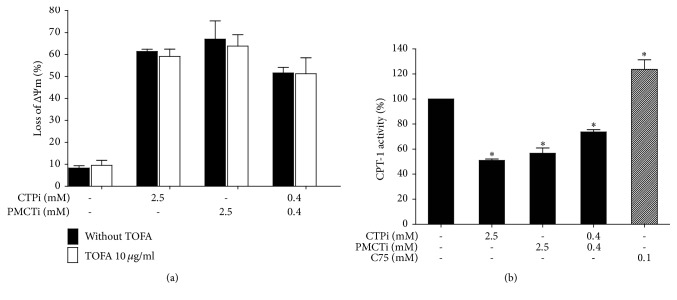
Effect of citrate transporter inhibitors on ΔΨm and activity of CPT-1 in HepG2 cells. (a) The loss of ΔΨm was determined by JC-1 staining and detected by flow cytometry. Cells were treated with 10 *μ*g/ml of TOFA for 24 h, pretreated with 10 *μ*g/ml of TOFA for 1 h followed by treatment with CTPi, PMCTi, and combined CTPi and PMCTi for 24 h. (b) Percentage of CPT-1 activity was determined in cells treated with each inhibitor and combined inhibitors and 0.1 mM of C75 for 24 h. The control was defined as a vehicle for which cells were treated with a medium containing 0.2% DMSO alone. Data are presented as mean ± SEM of three independent experiments, ^**∗**^*ρ* < 0.05.

We further investigated the accumulation of malonyl-CoA mediated by depletion of fatty acid synthesis as one of the major contributors to apoptotic induction by citrate transporter inhibitor [36, 37]. Fatty acid levels of the DNL pathway are known to exert a negative inhibitory effect on ACC activity that leads to alteration of malonyl-CoA synthesis, which in turn regulates CPT-1 activity [38, 39]. The present study showed that TOFA, an ACC inhibitor, treatment did not increase a loss of ΔΨm as compared with the control (Figure 7(a)). However, loss of ΔΨm as a result of CTPi, PMCTi, and the combination of 0.4 mM of each inhibitor treatment was not relieved following pretreating cells with TOFA, suggesting fatty acid itself contributes to suppression of CPT-1 activity-mediated mitochondrial damage. Additional supporting evidence found that in CTPi, PMCTi, and the combination of both inhibitors reduced CPT-1 activity by 50%, 44%, and 30%, respectively, while treatment with C75 increased CPT-1 activity to approximately 25%, compared with 100% of the vehicle control (Figure 7(b)). Taken together, these data suggest that apoptotic induction of citrate inhibitor treatment is correlated with depletion of fatty acid synthesis that leads to suppression of CPT-1 activity independent of an accumulation of malonyl-CoA level. Meanwhile, we found that TOFA did not restore apoptotic cells from cytotoxic effect of C75 because of a direct effect on upregulation of CPT-1 activity [40, 41].

### 3.8. Citrate Transporter Inhibitors Induced an Apoptosis Pathway through Decreased Bcl-2 Activity in HepG2 Cells

**Figure 8:**
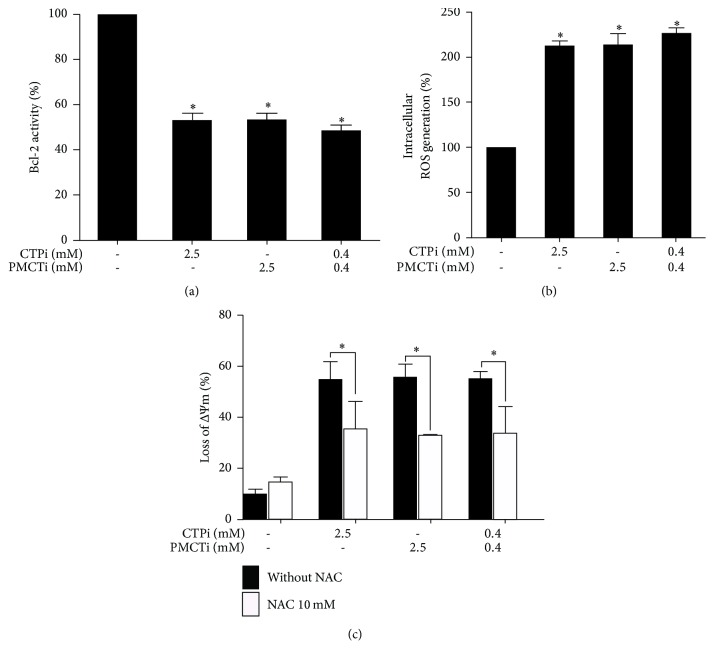
Effect of citrate transporter inhibitors on apoptosis induction and ROS generation in HepG2 cells. Cells were treated with CTPi, PMCTi, and a combination of each inhibitor for 24 h. (a) Bcl-2 activity was quantified by flow cytometry using Bcl-2 phosphorylation relative to total Bcl-2 expression levels compared with 100% of the control for which cells were treated with a medium containing 0.2% DMSO alone. (b) Intracellular ROS production was measured using flow cytometry. (c) Cells were pretreated with NAC 10 mM for 2 h and incubated with CTPi, PMCTi, and the combination for 24 h, and then ΔΨm was detected by flow cytometry. Data were presented as mean ± SEM, from at least three independent experiments, ^**∗**^*ρ* < 0.05.

Incubating cells with CTPi, PMCTi, and combined inhibitors for 24 h suppressed Bcl-2 activity to 52%, 53%, and 44%, respectively, compared with 100% of vehicle control (Figure 8(a)). Thus, citrate transporter inhibitors induce apoptosis through an intrinsic apoptotic pathway via suppression of Bcl-2 activity in HepG2 cells.

### 3.9. Citrate Transporter Inhibitors Induced Intracellular ROS Generation in HepG2 Cells

Increased intracellular generation of ROS has been reported to promote apoptosis in cancers [42, 43]. The present experiment found apoptotic induction by citrate transporter inhibitors was accompanied by an elevation of ROS formation. Cells treated with single treatment of 2.5 mM CTPi or PMCTi and a combination of 0.4 mM of each inhibitor significantly increased ROS production (Figure 8(b)). These results confirm that ROS plays a role in apoptotic induction following citrate transporter inhibitor treatment. N-acetylcysteine (NAC), a typical antioxidant, can scavenge free radicals and attenuate ROS, protecting HepG2 cell from loss of ΔΨm (Figure 8(c)). Thus, these results suggest that CTPi, PMCTi, and the combination of each inhibitor inducing apoptosis are related to increased ROS generation.

## 4. Discussion

The crucial role in aberrant fatty acid accumulation-induced cancer has been extensively studied since reducing* Indy* gene expression enhanced life longevity of lower organisms,* D. melanogaster* and* C. elegans*. This prolonged lifespan phenomenon resembles that which occurs by caloric restriction. Extended life span following* Indy* gene suppression or mutation is associated with decreased fat content [44]. The mechanism of which decreased cellular fatty acid level exhibits a beneficial effect has been proposed to involve the correcting of unfavourable consequencing metabolic disorders from fatty acid accumulation. The homolog of* Indy* found in mammalians* (mIndy)* is known to encode a plasma membrane NaCT and is expressed highest in the liver. Knockdown of* mIndy* provides similar evidence to support the importance of liver* mIndy*-NaCT citrate uptake inhibition in restricting hepatic triglyceride accumulation, thereby leading to the correction of insulin insensitivity [45]. Dentin et al. have reported and confirmed that elevated hepatic fatty acid accumulation is now identified as an important determinant cause of insulin resistance associated with obesity and type II diabetes [46]. Thus, elevation of de novo lipid synthesis manifested as enhanced citrate uptake has the importance of promoting unfavourable metabolic disorder consequences [47]. Recently, it has been established that molecular mechanisms are linked to a high risk of carcinogenesis development, especially hepatocellular carcinoma (HCC) and intracellular lipid accumulation in obesity. Tumor promoting-cytokines released from liver inflammatory response are responsible for tumor occurrence [28, 29]. Thus, the accumulation of fatty acid via de novo synthesis is proposed to result in enhanced risk of insulin insensitivity and cancer development. Taken together, these reports provide convincing evidence that inhibition of de novo lipid synthesis by reducing citrate uptake may, therefore, be a potential target for cancer prevention and therapy. However, the underlining mechanisms of inhibition of fatty acid accumulation leading to prevention of cancer development in the obese associated with the insulin resistant model have not been clearly established.

Birkenfeld has demonstrated that intracellular citrate level plays an important role as fuel sensing and signaling molecule in the regulation of de novo fatty acid synthesis and mitochondrial *β*-oxidation [45]. In addition to plasma membrane NaCT, citrate transported from CTP also supports cytosolic citrate to provide key substrate carbon sources from mitochondrial citric acid cycle intermediate for the DNL pathway. The overexpression of SLC25A1 or CTP is often found in many lipogenic tissues [48] while NaCT locates predominantly in the sinusoidal membrane of hepatocytes and moderately in spermatozoa, neuron, and salivary glands [49, 50], and a negative expression of NaCT has been reported in the small intestine [51]. Nowadays, high expression of these two transporter proteins in cancer cells shows important roles in cancer proliferation and progression [18, 52]. Although several studies have proposed important roles of citrate transporters as target treatments of metabolic disorders, there is little information of their transport roles for de novo lipid synthesis to facilitate the proliferation of cancer cells [53]. The expression level of CTP (or CiC) is positively correlated with lung cancer prognosis and chemotherapeutic drug resistance [20]. Surprisingly, the anti-tumor effect of suppressed CTP activity followed by a reduction of fatty acid synthesis level exhibits a selective cytotoxic effect on cancer cells, being nontoxic on normal adult tissues [54]. Taken together, they convincingly support the importance of the roles of citrate-derived fatty acid on cancer growth and proliferation. Based on these suggestions, there are several studies that have been extensively developed to suppress de novo fatty acid synthesis as a target for selective and effective cancer therapies in several cancer models [10–15]. Thus, suppression of DNL synthesis by targeting two major citrate transport pathways appears to be an attractive therapeutic strategy in cancers.

The effect of citrate transporter inhibition on the suppression of the DNL pathway contributing to apoptosis in hepatocellular carcinoma cells has not been well studied. We found more evident of synergistic cytotoxic effect of CTPi combined with PMCTi in HepG2 cells than in the HuH-7 cells. The present study demonstrated the effect of citrate transporter inhibitors targeted in abrogation of fatty acid production from the DNL pathway, which led to augmentation of mitochondrial-dependent apoptosis in HepG2 cells. Suppression of CPT-1 activity as a consequence of the depletion of fatty acid synthesis accounted for enhanced cytotoxic and apoptosis without involving an accumulation of malonyl-CoA. The present study showed a concomitant enhanced ROS generation secondary to decreased fatty acid production. The present study demonstrates new evidence of a synergistic antitumor efficacy of combined CTPi and PMCTi. We then, therefore, identified the mechanism of citrate transport inhibition regulated apoptosis in HepG2 cells. This approach may be a clinically available anticancer agent, supporting a direct translation of this combination strategy in the clinic for the selective treatment of hepatocellular carcinoma cells. It also supports the concept that combination of inhibition of fatty acid synthesis and genotoxic drugs or radiation reduces drug and radiation resistance by improving the DNA damage response pathway [55, 56].

Our observation showed that the activity of the lipogenic enzyme in the DNL pathway measured by intracellular fatty acid products, but not the expression of enzymes, including FASN, ACC, and ACLY, was suppressed following combined CTPi and PMCTi treatment for 24 h. This finding indicates a modulation of lipogenic enzyme activity instead of enzyme expression during a low substrate citrate level and hence confirms suppression of the DNL pathway. Our findings are consistent with results that previously reported that alteration of substrate availability does not influence in gene expression of enzymes in the long chain polyunsaturated fatty acid (LCPUFA) metabolism pathway [57]. Many previous studies have reported a cytotoxic effect of depleted LCFA level of the DNL pathway in enhancing apoptosis in cancer cells. Blockade of FASN leads to apoptosis in human breast cancer in vitro and in vivo [37] and FASN inhibition after epigallocatechin-3-gallate (EGCG) treatment induced apoptosis in breast cancer cells [36]. The effect of siRNA-mediated knockdown of ACC-*α* was observed on growth arrest and tumor cell death in prostate cancer cells [58]. Inhibition of ACLY causes the growth suppression of the prostate cancer cell line [59]. Decreased fatty acids and triglycerides induce alteration of lipid composition that switches the cancer cells from proliferation to quiescent state in the lung, glioblastoma, astrocytoma, and leukemia cancer cell [60].

Enhanced ROS generation has been recognized as one of the causes of apoptosis with mitochondrial dysfunction in human cancer cells [43, 61]. In the present study, we found that citrate transporter inhibitor-induced apoptosis through the loss of ΔΨm was related to an increased ROS level. We suggest that the combination of CTP and PMCT inhibitors targets ROS generation that is also considered to trigger apoptosis in HepG2 cells. This finding indicates that suppression of fatty acid synthesis as a result of inhibition of citrate transport contributes to accelerating ROS generation. Previous studies have reported the effect of depleted de novo fatty acid synthesis on enhanced ROS generation in many cancer cells by various inhibitors of the DNL pathway, including cerulenin and orlistat [62, 63]. ACLY suppression by ACLY knockdown induced an anticancer effect via reactive oxygen species in many cancer cells [63]. However, the molecular mechanism of fatty acid suppression regarding enhanced ROS generation has not been fully identified. Noticeably, a recent study has reported that mitochondrial citrate accumulation to suppress succinate dehydrogenase (SDH) is considered to regulate ROS production in rat liver mitochondria [64, 65]. Our findings showed a decreased intracellular citrate level by the action of combined CTPi and PMCTi, implying an accumulation of citrate from the TCA cycle in mitochondria. The intramitochondrial citrate directly inhibits complex II or succinate-ubiquinone oxidoreductase, commonly known as SDH, a tetrameric iron-sulfur flavoprotein of the inner mitochondrial membrane that acts as the catalyzer of conversion of succinate into fumarate [65]. Impaired electron transport as a result of SDH inhibition promotes a substantial amount of ROS production leading to apoptosis in human HeLa cells [66]. Increased ROS level has been proposed to cause apoptosis via suppression of Bcl-2 expression, activating cytochrome c, caspase 9, and caspase 3 expression [67–69].

In addition to LCFA, sphingolipids such as ceramide and sphingosine-1-phosphate (S1P) are generated from fatty acid product of the DNL pathway. Sphingolipids are synthesized from the condensation of palmitate, which is one of the most productive LCFAs and serine to form dihydrosphingosine. Dihydroceramide is transformed from dihydrosphingosine by the activity of (dihydro)-ceramide synthase. Ceramide is finally generated from dihydroceramide by the action of the desaturase enzyme. Ceramide can be concomitantly converted to other interconnected bioactive lipid species, especially S1P, by sphingosine kinase (SphKs) [70]. Furthermore, balancing roles between sphingolipids, ceramide, and S1P are required for cell function and survival. It has been reported that ceramide induces apoptosis in cancer cells while S1P functions as a survival factor in various cells [71]. The current evidence has demonstrated that antiapoptotic Bcl-2 reduces ceramide accumulation and the mitochondrial permeability transition pore opening as well as increases intracellular S1P levels by stimulating the expression and activity of SphK1, thereby decreasing the ceramide/S1P ratio [72]. In addition, Bcl-2 affects the enzymes involved in ceramide metabolisms, such as ceramide synthases and sphingomyelinases, leading to a reduction of apoptosis and increase of cell survival [73, 74]. The present study found that citrate transporter inhibitors caused suppression of Bcl-2 activity. Thus, we suggest that suppression of Bcl-2 activity-mediated by ROS production as a consequent of inhibition of mitochondrial citrate transport enhances ceramide accumulation that leads to the promotion of apoptosis in HepG2 cells.

The previous report has suggested that the transcriptional factor PPAR*α* is one of the regulators of CPT-1 expression. Depletion of ACLY or LCFAs has been reported as suppressors of PPAR*α* activity [59] which then targets a reduction of CPT-1 activity. Such reduction leads to suppression of the delivery rate of LCFAs into mitochondria for fatty acid *β*-oxidation. In particular, this suppression consequently accounts for cytosolic LCFA and fatty acyl-CoA accumulation that provide a stimulatory effect on ceramide production [75, 76]. Those regulatory pathways on CPT-1 activity were shown to be consistently observed in the present study describing the reduction of CPT-1 activity in the presence of a low level of malonyl-CoA production. Thus, we suggest that suppression of LCFA following an applied combination of citrate transporter inhibitors downregulates CPT-1 activity through depletion of PPAR*α*. Those effects indicate that catalization and transferration of LCFAs for the fatty acid *β*-oxidation process are abolished. Taken together, accumulation of LCFAs can be converted to ceramide which triggers cellular consequences of apoptosis. Increased expressions of pro-apoptotic genes BNIP3, tumor necrosis factor related apoptosis-inducing ligand (TRAIL), and death-associated protein kinase 2 (DAPK2) have been reported to contribute to apoptosis induction in ceramide accumulation in cancer cells [77]. However, further studies are needed to address the role of LCFAs on ceramide production-induced apoptosis in cancer cells. In addition to this evidence, a recent study reports that inhibition of liver form-CPT-1A activity suppresses cell proliferation and induces cell cycle arrest in ovarian cancer cells. Suppressed CPT-1A causes ATP depletion from fatty acid *β*-oxidation that leads to activating cell cycle progression controlling pathways: AMPK, JNK, and p38 kinase pathway [78].

## 5. Conclusions

In conclusion, our findings demonstrate that the combination of CTP and PMCT inhibitor will potentially provide an attractive development of a novel anti-cancer therapy that targets the inhibition of DNL for HCC and other cancers. The ability to selectively inhibit the DNL pathway may be a potential therapeutic to relieve unfavorable metabolic disorders of obesity and prevent the cancer occurrence consequence since these aberrant metabolic regulations are known to be associated with an excessive synthesis of fatty acid. Further study targeting the citrate transporter protein may be useful to prevent hepatic insulin resistance and cancer as a result of declined mitochondrial function-induced abnormality in intracellular lipid accumulation in aging.

## List of Abbreviations

CTP: Mitochondrial citrate transporter
PMCT: Plasma membrane citrate transporter protein
CPT-1: Carnitine palmitoyl transferase-1
DNL: De novo lipogenesis
HCC: Hepatocellular carcinoma
OXPHOS: Oxidative phosphorylation
LCFAs: Long chain fatty acids
ACLY: ATP-citrate lyase
ACC: Acetyl-CoA carboxylase
FASN: Fatty acid synthase
MUFAs: Monounsaturated fatty acids
ROS: Reactive oxygen species
TOFA: 5-(Tetradecyloxy)-2-furoic acid
CCCP: Carbonyl cyanide m-chlorophenylhydrazone.
